# Gut microbiota and their putative metabolic functions in fragmented Bengal tiger population of Nepal

**DOI:** 10.1371/journal.pone.0221868

**Published:** 2019-08-29

**Authors:** Dibesh Karmacharya, Prajwol Manandhar, Sulochana Manandhar, Adarsh M. Sherchan, Ajay N. Sharma, Jyoti Joshi, Manisha Bista, Shailendra Bajracharya, Nagendra P. Awasthi, Netra Sharma, Bronwyn Llewellyn, Lisette P. Waits, Kanchan Thapa, Marcella J. Kelly, Momchilo Vuyisich, Shawn R. Starkenburg, Jean-Marc Hero, Jane Hughes, Claudia Wultsch, Laura Bertola, Nicholas M. Fountain-Jones, Amit K. Sinha

**Affiliations:** 1 Center for Molecular Dynamics Nepal, Kathmandu, Nepal; 2 School of Environment, Griffith University, Brisbane, Queensland, Australia; 3 Environment Team, U.S. Agency for International Development, Kathmandu, Nepal; 4 Department of Fish and Wildlife Sciences, University of Idaho, Moscow, Idaho, United States of America; 5 Department of Fish and Wildlife Conservation, Virginia Tech, Blacksburg, Virginia, United States of America; 6 Applied Genomics, Los Alamos National Lab, Los Alamos, New Mexico, United States of America; 7 School of Science & Education, University of the Sunshine Coast, Sunshine Coast, Queensland, Australia; 8 Sackler Institute for Comparative Genomics, American Museum of Natural History, New York, United States of America; 9 Bioinformatics and Computational Genomics Laboratory, Hunter College, City University of New York, New York, United States of America; 10 Department of Biology, City College of New York, New York, United States of America; 11 Institute of Environmental Sciences, Leiden University, Leiden, The Netherlands; 12 Department of Veterinary Population Medicine, University of Minnesota, Minneapolis, Minnesota, United States of America; University of Oregon, UNITED STATES

## Abstract

Bengal tigers (*Panthera tigris tigris*) serve a pivotal role as an apex predator in forest ecosystems. To increase our knowledge on factors impacting the viability and health of this endangered species, we studied the gut microbiota in 32 individual Bengal tigers from three geographically separated areas (Chitwan National Park (CNP), Bardia National Park (BNP) and Suklaphanta Wildlife Reserve (SWR)) in Nepal, using noninvasive genetic sampling methods. Gut microbiota influence the immune system, impact various physiological functions, and modulates metabolic reactions, that ultimately impact the host health, behavior and development. Across the tiger populations in Nepal, we found significant differences in the composition of microbial communities based on their geographic locations. Specifically, we detected significant differences between CNP and the other two protected areas (CNP vs BNP: pseudo t = 1.944, *P* = 0.006; CNP vs SWR: pseudo t = 1.9942, *P* = 0.0071), but no differences between BNP and SWR. This mirrors what has been found for tiger gene flow in the same populations, suggesting gut microbiota composition and host gene flow may be linked. Furthermore, predictive metagenome functional content analysis (PICRUSt) revealed a higher functional enrichment and diversity for significant gut microbiota in the Chitwan tiger population and the lowest enrichment and diversity in Suklaphanta. The CNP tiger population contained higher proportions of microbiota that are associated with predicted functions relevant for metabolism of amino acid, lipid, xenobiotics biodegradation, terpenoides and polyketides than the SWR population. We conclude the tiger population structure, gut microbiota profile and associated functional metabolic categories are correlated, with geographically most separated CNP and SWR tiger population having the most distinct and different host genotype and microbiota profiles. Our work dramatically expands the understanding of tiger microbiota in wild populations and provides a valuable case study on how to investigate genetic diversity at different hierarchical levels, including hosts as well as their microbial communities.

## Introduction

Gut microbiota are a complex community of microorganisms in the intestinal tract that has co-evolved with the host [1, 2] playing an important role in maintaining the host’s health. Gut microbial communities shape the immune system, impact various physiological functions, and modulate metabolic reactions that ultimately impact the host health, fitness, behavior, digestion and development [3–5]. The composition of gut microbiota are largely determined by several intrinsic and extrinsic factors such as the host’s environment, health status, genotype, dietary habits, age, sex, social relationships and disease prevalence [6–10]. For example, the composition of gut microbiota in wildlife may change in response to anthropogenic stresses such as the loss and fragmentation of host habitat [11–14]. Habitat fragmentation could alter the microbiota directly via changes in diet and/or exposure to human associated microbes [15] or indirectly via changes in host genetic structure [16, 17]. The indirect effects of host genetics on gut microbial community structure or ‘phylosymbiosis’ are poorly understood in wild animal populations and are likely interact with other factors such as shifts in diet [17]. Untangling the relative importance of these direct or indirect effects is difficult in wild animal populations (i.e, different populations have different diets) but crucial given the importance of the gut microbiota to the health of individuals and populations.

In the last decade, spurred by technological advances in DNA sequencing, multiple studies have described gut microbiota of various terrestrial and aquatic species, including humans, primates, whales and other mammals [18–20]. Gut microbiome composition is critical for the host’s health and disturbances in the bacterial microbiota might, for example, result in immunological dysregulation that may underlie disorders such as inflammatory bowel disease, Crohn's disease, and ulcerative colitis [21, 22]. The mammalian immune system which appears to control microbes, in fact, might be controlled by the microbes themselves [23]. For example, by stimulating the immune system and the development of gut structure, gut microbes play a crucial role in the regulation of host health by aiding in the defense against invading pathogens and providing nutritional benefit to the host such as the production of short chain fatty acids and vitamin B12 [24]. As microbial communities inhabiting wildlife species greatly affect host health, nutrition, physiology and immune systems, understanding gut microbial community dynamics is increasingly considered crucial for successful wildlife conservation and management programs [7, 25, 26].

Globally, the population of wild tigers is declining dramatically due to widespread habitat loss and fragmentation, prey depletion, illegal hunting and various infectious diseases [27–30]. Within Nepal, habitat loss and fragmentation have forced extant tigers to divide into distinct geographically separated populations in Nepal, which has been extensively studied using long-term field data [31] and noninvasive genetic sampling [32]. The Bengal tiger’s main habitat in Nepal is restricted to five protected areas along the Terai Arc Landscape (TAL), including Chitwan National Park (CNP), Parsa Wildlife Reserve (PWR), Bardia National Park (BNP), Banke National Park (BaNP), and Suklaphanta Wildlife Reserve (SWR) [33, 34]. The TAL has experienced significant land use changes in the recent past [28, 34]. Human settlements surround and encroach into tiger habitat degrading natural areas and potentially increasing levels of environmental stress for tigers. This region has also experienced severe socio-political unrest, which included 10 year civil war during the Maoist insurgency, that has negatively impacted fragile ecosystems with weakened wildlife conservation programs [35, 36]. Considering the degree of environmental degradation in conjunction with habitat loss and fragmentation over the last century [32], it is vital to take a multidimensional and interdisciplinary approach to monitoring and managing the health of wild tiger subpopulations.

**Fig 1:**
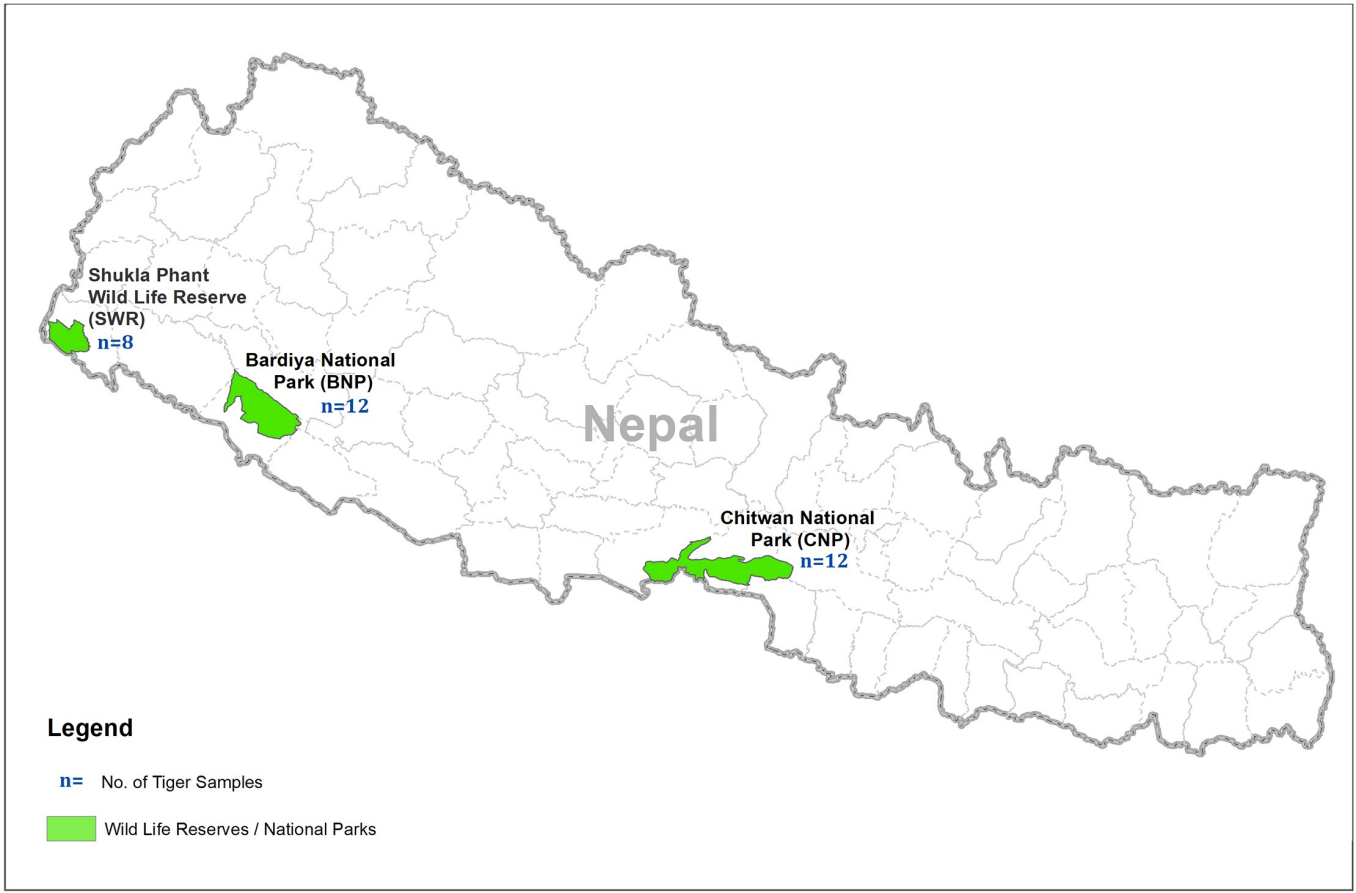
Scat sample collection sites for tiger baseline genetic database under Nepal Tiger Genome Project (NTGP, 2011–2013). We identified 120 individual tigers using 8 microsatellite markers from TAL (SWR = 19, BNP = 32, CNP = 69). A total of 70 tiger scat samples from 32 identified individual tigers (CNP = 12; BNP = 12; SWR = 8) were randomly selected for gut microbiota analysis.

The extent to which habitat loss and fragmentation alter the gut microbiota and in turn impact the health of endangered wildlife is largely unknown. Small isolated wildlife populations may not only have low genetic diversity but also have a low gut microbial diversity with an altered functionality that could adversely impact the health of these animals and potentially increase the risk of local extinction [17]. For example, the Red colobus monkey (*Procolobus gordonorum*), an endangered species, residing around human settlements seemed to have reduced gut microbial diversity compared to a population found in a wild habitat [12]. In our previous work, we identified 120 individual tigers based on field-collected fecal samples using eight microsatellite markers and found that tigers in SWR had the lowest genetic diversity and were the most isolated in terms of gene flow compared to the other parks. Tigers from CNP and BNP had similar levels of genetic diversity even though CNP is geographically distant from BNP and SWR [32]. Based on this, we hypothesized that SWR might have the least diverse microbiota compared to the other tiger populations. However, as anthropogenic effects can substantially perturb the microbiota of wildlife [37], we expect that tigers in the CNP may have a unique gut microbiome composition as this park receives much higher human visitation compared to the other parks. The aim of our study is to examine structural and functional diversity of gut microbial communities in tiger populations of TAL (Fig 1).

As part of the Nepal Tiger Genome Project (NTGP) [38], we conducted one of the largest microbiota surveys of a wild carnivore spanning three populations with different degrees of connectivity and human visitation. We take advantage of data of likely prey species to help untangle the drivers of microbial community structure and assess what role phylosymbiosis plays in structuring the tiger microbiota. This study increased our knowledge of tiger gut microbiota and the information could contribute towards the development of a more comprehensive strategy to conserve and manage wild tiger populations occurring across fragmented landscapes.

## Results

### Composition of tiger gut microbiota across different protected sites of Nepal

**Fig 2:**
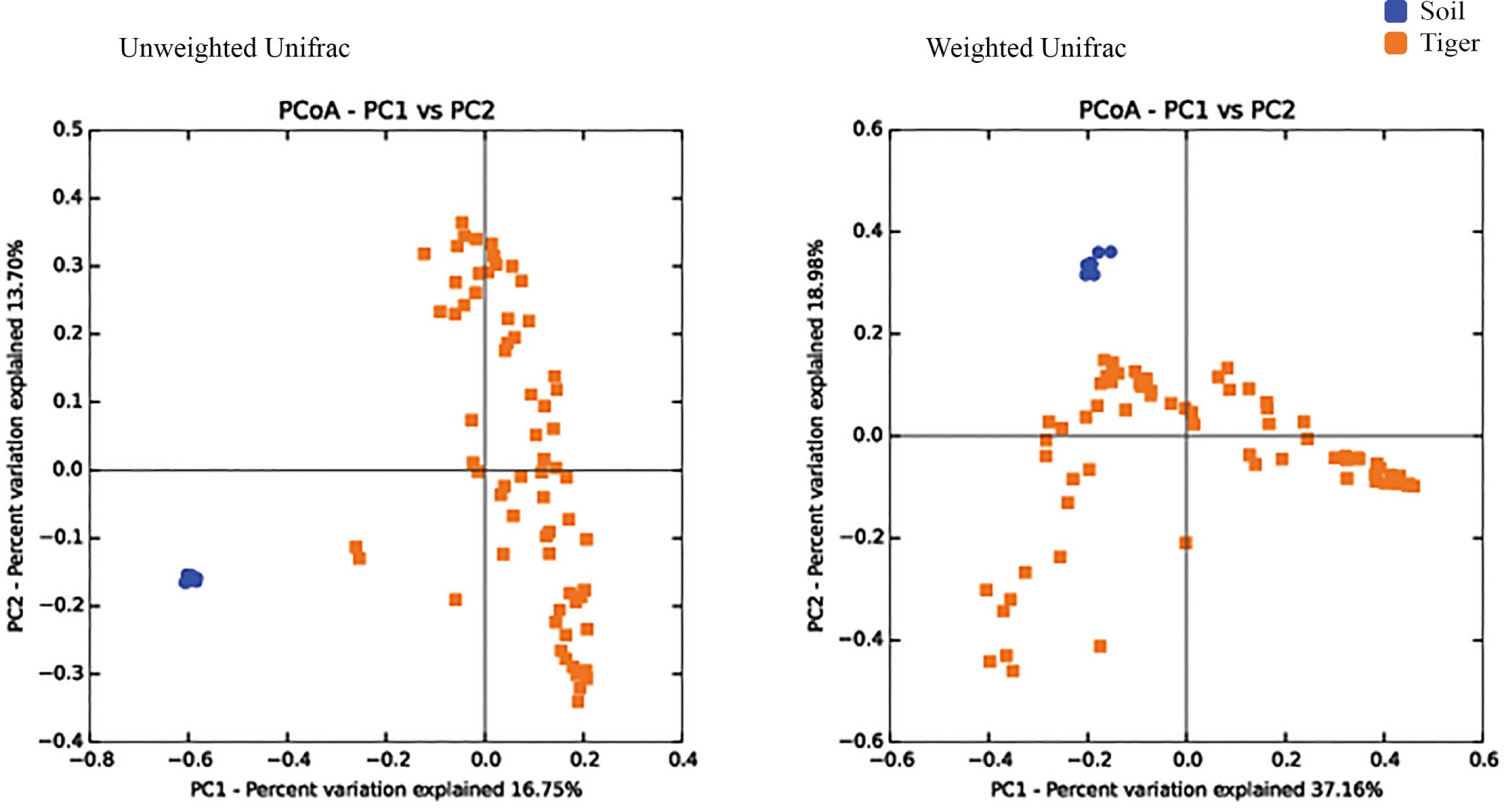
Principal Coordinate Analysis (PCoA) of soil (all sites, *n* = 8; CNP, *n* = 3; BNP, *n* = 5) and tiger fecal samples (all sites, *n* = 32; CNP, *n* = 12; BNP, *n* = 12; SWR, *n* = 8). Gut microbiota profiles for soil samples are distinct from fecal samples indicating that cross-contamination between these two sample sources is unlikely.

**Fig 3:**
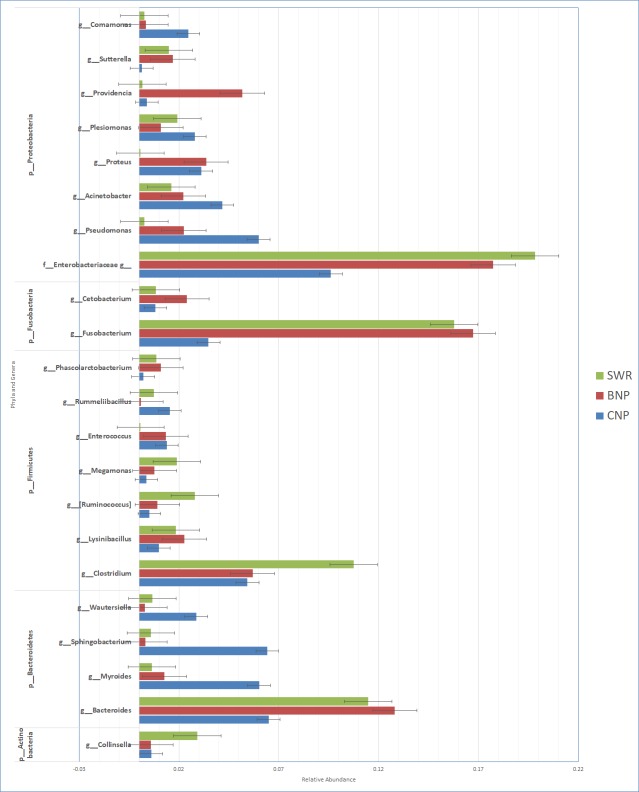
Gut microbiota diversity in tiger populations of Nepal. Relative abundance of top five microbial phyla and their subsequent genera identified in tiger fecal samples collected across three protected areas (CNP, BNP, SWR) within TAL.

**Fig 4:**
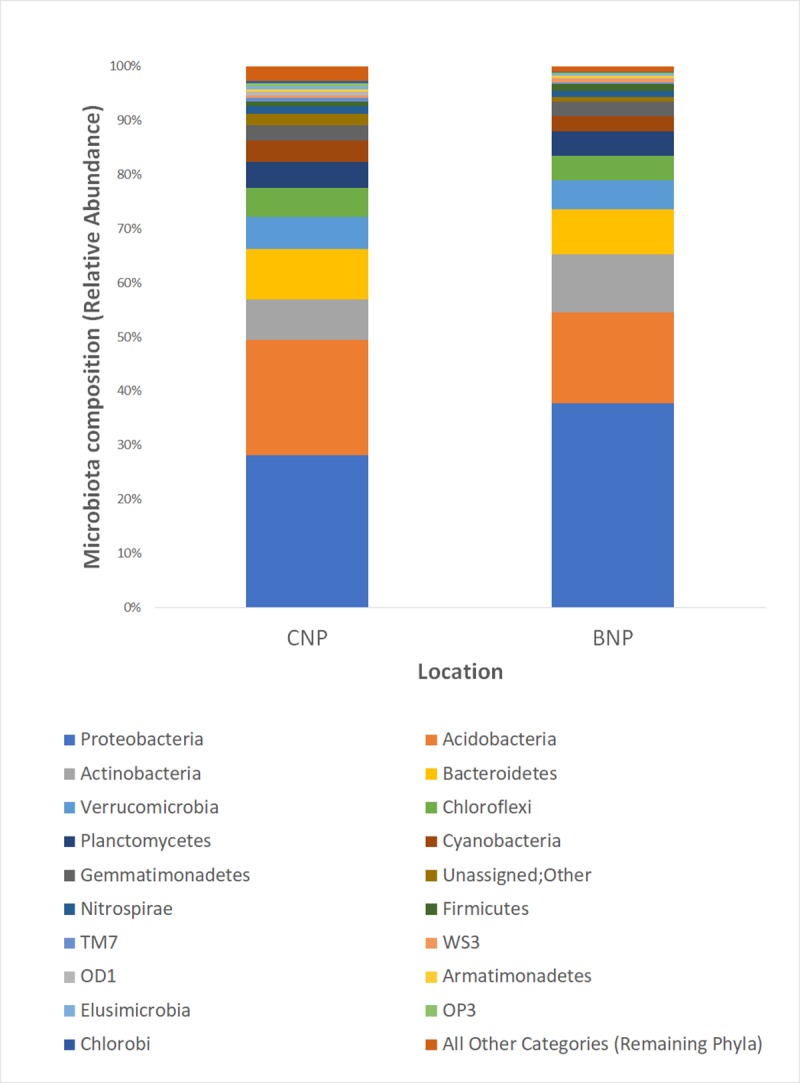
Soil microbiota biodiversity examined at two TAL sites. Relative abundance of microbial phyla detected in soil samples (*n* = 8) collected at CNP (*n* = 3) and BNP (*n* = 5).

16S rRNA amplicon sequencing of tiger scat and soil samples (tiger, *n* = 70; soil, *n* = 8) targeting the hypervariable V4 region of 16S rRNA gene generated a total of 4,385,688 sequences, among which tiger samples consisted of 2,985,814 sequences and soil samples consisted of 1,399,874 sequences. For 70 tiger samples from 32 individual tigers, the mean number of sequences per sample was 42,654 (range: 1,614–95,553). Similarly, for 8 soil samples, the mean number of sequences per sample was 174,984 (range: 134,283–235,580). After rarefaction, four samples having less than 10,000 sequences were filtered out and excluded from further analyses (S1 Table and S1 Fig). The gut microbiota communities characterized via Operational Taxonomic Unit (OTU), with ≥ 97% nucleotide sequence identity, differed significantly between soil and tiger samples (PERMANOVA; Unweighted Unifrac: pseudo F = 12.69, *P* = 0.001; Weighted Unifrac: pseudo F = 15.52, *P* = 0.001) (Fig 2). Overall composition of highly abundant microbiota of tiger samples are very similar across the three regions (Fig 3). Overall, the most dominant phyla detected in the gut microbiota of tigers were Proteobacteria (37.1% +/- 8.49E-02), Firmicutes (30.1% +/- 8.54E-02), Bacteroidetes (16.1% +/- 5.48E-02), Fusobacteria (12.3% +/- 6.47E-02), and Actinobacteria (2.8% +/- 1.40E-02) (Table 1 and Fig 3). The major microbial phyla present in soil were Proteobacteria (33% +/- 6.9E-02), Acidobacteria (19% +/- 3.2E-02), Actinobacteria (9% +/- 2.3E-02) and Bacteroidetes (9% +/- 7.8E-03) (Table 2 and Fig 4). Acidobacteria, Verrucomicrobia, Chloroflexi, Planctomycetes, Cyanobacteria and Gemmatimonadetes are only observed in soil samples (Table 2). Fusobacteria were only found in tiger samples. Bacteroidetes, Firmicutes, Proteobacteria and Actinobacteria are common in both soil and tiger samples (Tables 1 and 2). We also compared gut microbiota profile between samples of same individuals (*n* = 6) collected at various times and observed slightly different microbiota profiles of identified phyla (S2–S7 Figs).

**Table 1:** Relative abundance of gut microbiota composition in Bengal tigers across three protected areas within TAL. The 10 most abundant bacterial phyla detected in tiger fecal samples collected across three major protected sites (CNP, BNP, SWR) within the Terai Arc Landscape of Nepal.

Phylum	CNP (Stdev)	BNP (Stdev)	SWR (Stdev)	Total (Mean +/- error)
Proteobacteria	40.5% (2.97E-01)	43.3% (3.55E-01)	27.4% (3.01E-01)	37.1% (8.49E-02)
Firmicutes	27.8% (2.85E-01)	22.9% (2.11E-01)	39.5% (2.38E-01)	30.1% (8.54E-02)
Bacteroidetes	22.4% (2.19E-01)	12.8% (1.46E-01)	13.1% (1.30E-01)	16.1% (5.48E-02)
Fusobacteria	4.9% (6.86E-02)	16.8% (1.75E-01)	15.2% (1.53E-01)	12.3% (6.47E-02)
Actinobacteria	2.8% (4.16E-02)	1.4% (2.50E-02)	4.2% (5.61E-02)	2.8% (1.40E-02)
Tenericutes	1.3% (7.03E-02)	2.2% (8.96E-02)	0.1% (2.07E-03)	1.2% (1.03E-02)
TM7	0.03% (6.05E-04)	0.5% (1.87E-02)	0.2% (4.01E-03)	0.2% (2.09E-03)
Unassigned;Other	0.1% (1.20E-03)	0.1% (2.23E-03)	0.1% (5.53E-04)	0.1% (2.48E-04)
Verrucomicrobia	0.1% (4.05E-03)	0.004% (9.05E-05)	0.01% (2.94E-04)	0.04% (5.51E-04)
All Other Categories (Remaining Phyla)	0.001% (3.86E-05)	0.002% (5.20E-05)	0.01% (1.43E-04)	0.003% (2.15E-05)

**Table 2:** Relative abundance of microbiota composition of soil samples collected at two protected areas (all sites *n* = 8; CNP, *n* = 3; BNP, *n* = 5) within TAL.

Phylum	CNP (Stdev)	BNP (Stdev)	Total (Mean +/- error)
Proteobacteria	28.1% (1.11E-01)	37.8% (4.34E-02)	32.9% (6.86E-02)
Acidobacteria	21.3% (9.64E-02)	16.8% (2.47E-02)	19.1% (3.21E-02)
Actinobacteria	7.5% (1.78E-02)	10.8% (5.03E-02)	9.1% (2.30E-02)
Bacteroidetes	9.4% (3.27E-02)	8.3% (2.29E-02)	8.8% (7.80E-03)
Verrucomicrobia	5.9% (8.12E-03)	5.4% (1.15E-02)	5.6% (3.48E-03)
Chloroflexi	5.4% (7.45E-03)	4.5% (3.68E-03)	4.9% (6.35E-03)
Planctomycetes	4.8% (8.67E-03)	4.5% (7.45E-03)	4.6% (2.60E-03)
Cyanobacteria	3.9% (5.10E-02)	2.8% (1.81E-02)	3.4% (8.04E-03)
Gemmatimonadetes	2.7% (8.81E-04)	2.8% (4.57E-03)	2.8% (3.36E-04)
Unassigned;Other	2.2% (3.95E-03)	0.8% (6.49E-03)	1.5% (9.89E-03)
Nitrospirae	1.4% (5.58E-03)	1.2% (5.51E-03)	1.3% (1.78E-03)
Firmicutes	0.7% (5.45E-03)	1.2% (7.00E-03)	1.0% (3.33E-03)
TM7	0.8% (7.47E-03)	0.3% (4.83E-04)	0.6% (3.49E-03)
WS3	0.3% (2.40E-03)	0.7% (3.05E-03)	0.5% (2.47E-03)
OD1	0.7% (4.75E-03)	0.2% (3.40E-03)	0.5% (3.60E-03)
Armatimonadetes	0.5% (4.12E-03)	0.3% (1.11E-03)	0.4% (1.41E-03)
Elusimicrobia	0.6% (4.49E-03)	0.3% (1.27E-03)	0.4% (1.83E-03)
OP3	0.5% (3.46E-03)	0.3% (3.15E-03)	0.4% (2.05E-03)
Chlorobi	0.4% (1.61E-03)	0.2% (1.29E-03)	0.3% (1.32E-03)
All Other Categories (Remaining Phyla)	2.7% (1.61E-02)	1.0% (9.05E-03)	1.8% (1.23E-02)

All the raw sequences associated with this study have been deposited at figshare repository and can be publicly assessed using the link: **https://doi.org/10.6084/m9.figshare.8010389.v1**

### Alpha-diversity (within population) measures of tiger gut microbiota

**Fig 5:**
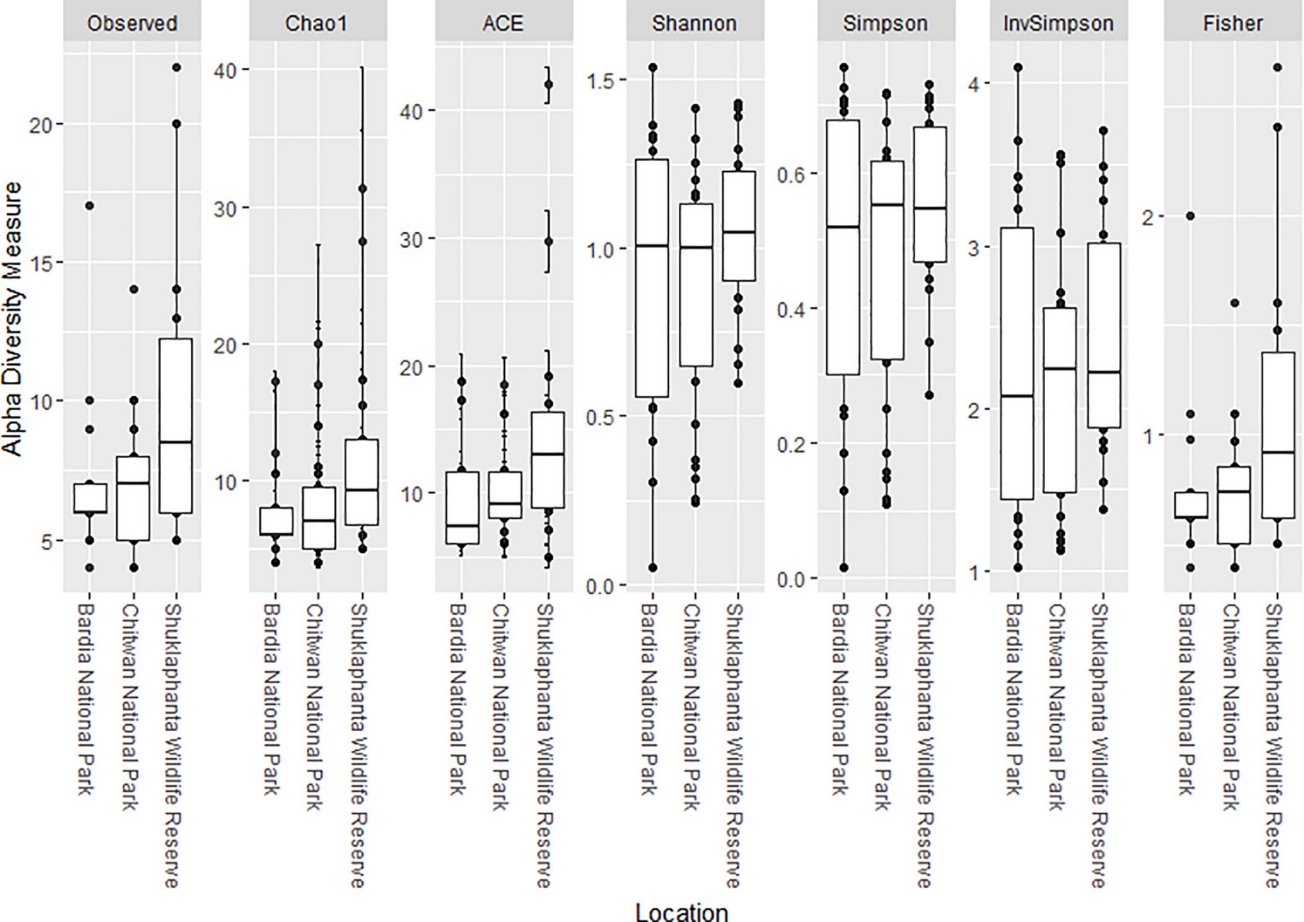
Alpha-diversity in tiger gut microbial communities is similar across different regions studied.

Overall, there was no significant difference in alpha diversity (within population) assessed in fecal microbiota samples collected from three tiger populations independent of the index used (Chao1 and ACE metrics Shannon’s index, Simpson’s index [39], Inverted Simpson and Fisher’s indexes [40]) (Fig 5).

### Beta-diversity (between population) of the tiger gut microbiota

**Fig 6:**
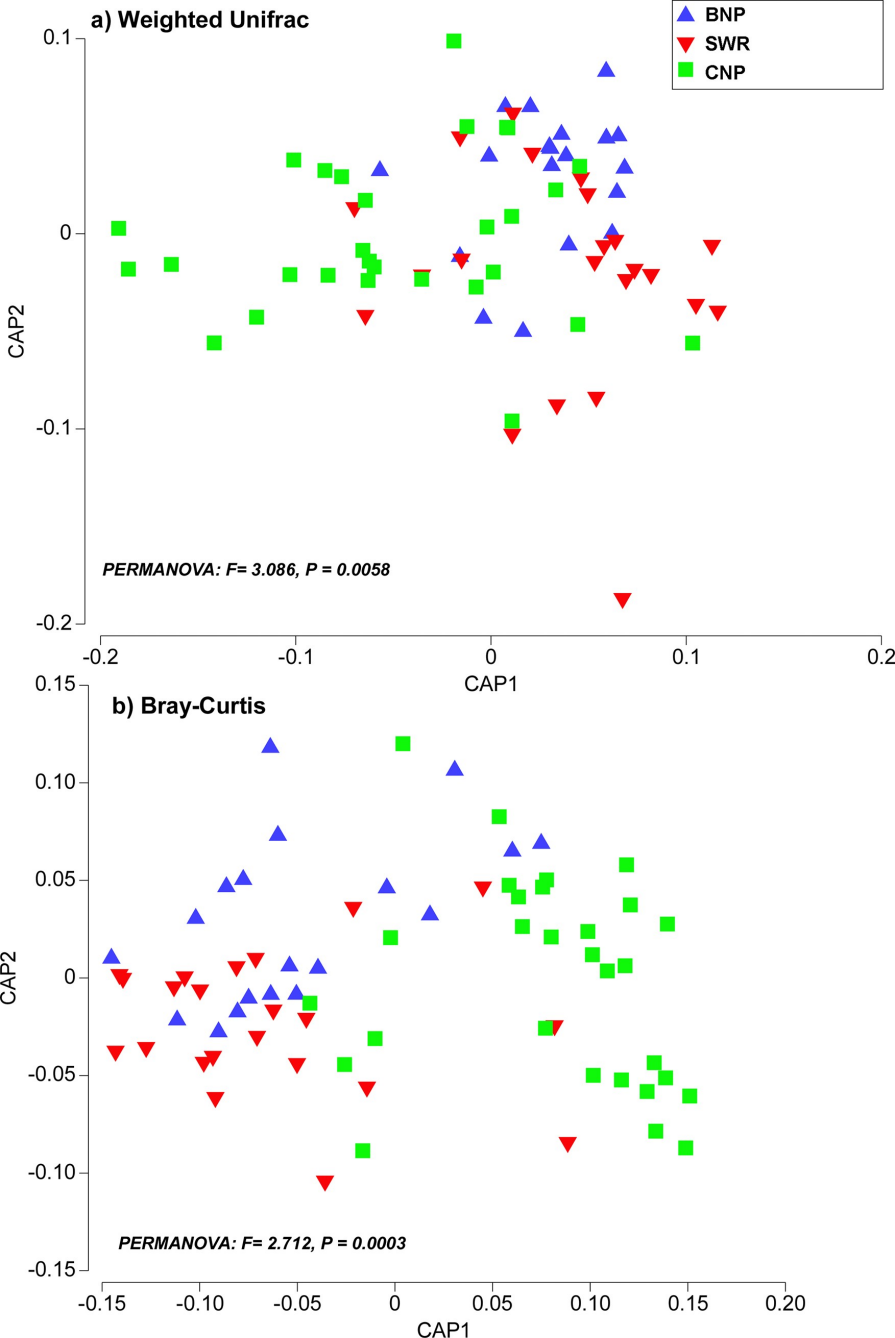
Canonical analysis of principal (CAP) coordinates showing differences in microbiota composition across three protected sites within TAL. PERMANOVA of both phylogenetic composition (weighted UniFrac distance) (a) and taxonomic composition (Bray-Curtis similarity) (b) similarity measures illustrated that CNP had differing microbial composition and overall greater beta diversity.

Our beta diversity analysis (between population) revealed significant differences in the phylogenetic and taxonomic composition of microbial communities across the tiger populations. We compared both phylogenetic and taxonomic diversity to test if differences between microbial beta-diversity across these tiger populations were driven by evolutionary history (i.e., different lineages present) or taxonomic membership (i.e., similar lineages present in each community, but different species detected, as described in [41] for the utility of comparing both diversity measures). Canonical analysis of principal coordinates (CAP) of weighted UniFrac distances indicated that microbial communities clustered based on their geographic locations with moderate levels of overlap (Fig 6). One-way PERMANOVA on weighted UniFrac distances found that the clustering across all areas was significant (pseudo F = 3.086, *P* = 0.006) with pairwise tests showing significant differences between CNP and the other two protected areas (CNP vs BNP: pseudo t = 1.944, *P* = 0.006; CNP vs SWR: pseudo t = 1.994, *P* = 0.007), but no significant differences between SWR and BNP (t = 0.782, *P* = 0.606) (Fig 6A). Furthermore, there was a greater beta phylogenetic diversity between CNP compared to SWR (PERMDISP, pseudo t = 2.72, *P* = 0.015), but not between CNP and BNP (PERMDISP, pseudo t = 1.92, *P* = 0.083) or BNP and SWR (PERMDISP, pseudo t = 0.79, *P* = 0.797). CAP confirmed these results by correctly assigning samples to each respective group most of the time (57%; null allocation success = 0.33) (Fig 6A).

We found similar patterns when we analyzed taxonomic beta-diversity based on Bray-Curtis similarity with significant clustering of tiger populations (PERMANOVA, pseudo F = 2.712, *P* = 0.0003) and similar pair-wise contrasts (CNP vs SWR: pseudo t = 2.056, *P* = 0.003; CNP vs BNP: t = 1.512, *P* = 0.005; BNP vs SWR: pseudo t = 1.114, *P* = 0.216). CNP had higher taxonomic beta-diversity than other protected sites studied here (PERMDISP, CNP vs BNP, pseudo t = 2.36, *P* = 0.031, CNP vs SWR: pseudo t = 3.511, *P* = 0.001), however, there was no difference in beta-diversity between BNP or SWR (PERMDISP, pseudo t = 1.21, *P* = 0.27). CAP correctly assigned samples to each population in most cases (63%, null allocation success = 0.33) (Fig 6B).

### Statistical analysis for bacterial abundance in tiger fecal microbiota

Analysis of differences in abundance, based on the F test (with Benjamini and Hochberg control for false discovery rate [42]) identified significant differences in three bacterial phyla (Fusobacteria, TM7 and Thermi) across the three protected sites. Phyla Fusobacteria and TM7 separated samples collected in CNP from a combined group of samples from BNP and SWR (adjusted *P* = 0.01, fdr = 0.028). The abundance of the phylum Thermi, also referred to as Deinococcus-Thermus, was significantly different in SWR samples from samples collected in BNP and CNP (adjusted *P* = 0.072, fdr = 0.168).

This inference motivated a more in-depth analysis with representative sequences obtained for twenty of the most prevalent genera (based on presence/absence). A statistical analysis with F-test and a Benjamini and Hochberg correction for false discovery identified significant differences in *Comamonas*, *Collinsella*, and *Fusobacterium* across CNP, BNP, and SWR with significant adjusted *P* values and acceptable rates of false discovery (fdr) (Table 3).

**Table 3:** Statistical analysis for most prevalent microbial phyla and genera in gut microbiota found in three sub-populations of tigers in Nepal.

Phylum	Genus	Relative Abundance (StDev)			Adj *P*	False discovery rate (fdr)
		CNP	BNP	SWR		
Proteobacteria	*Comamonas*	2.47% (2.68E-02)	0.36% (1.99E-02)	0.26% (7.89E-03)	0.03	0.024
Actinobacteria	*Collinsella*	0.61% (2.32E-02)	0.61% (9.05E-03)	2.93% (5.22E-02)	0.03	0.024
Fusobacteria	*Fusobacterium*	3.49% (6.37E-02)	16.73% (1.66E-01)	15.79% (1.51E-01)	0.109	0.064

### Predictive metabolic functions associated with tiger gut microbiota

**Fig 7:**
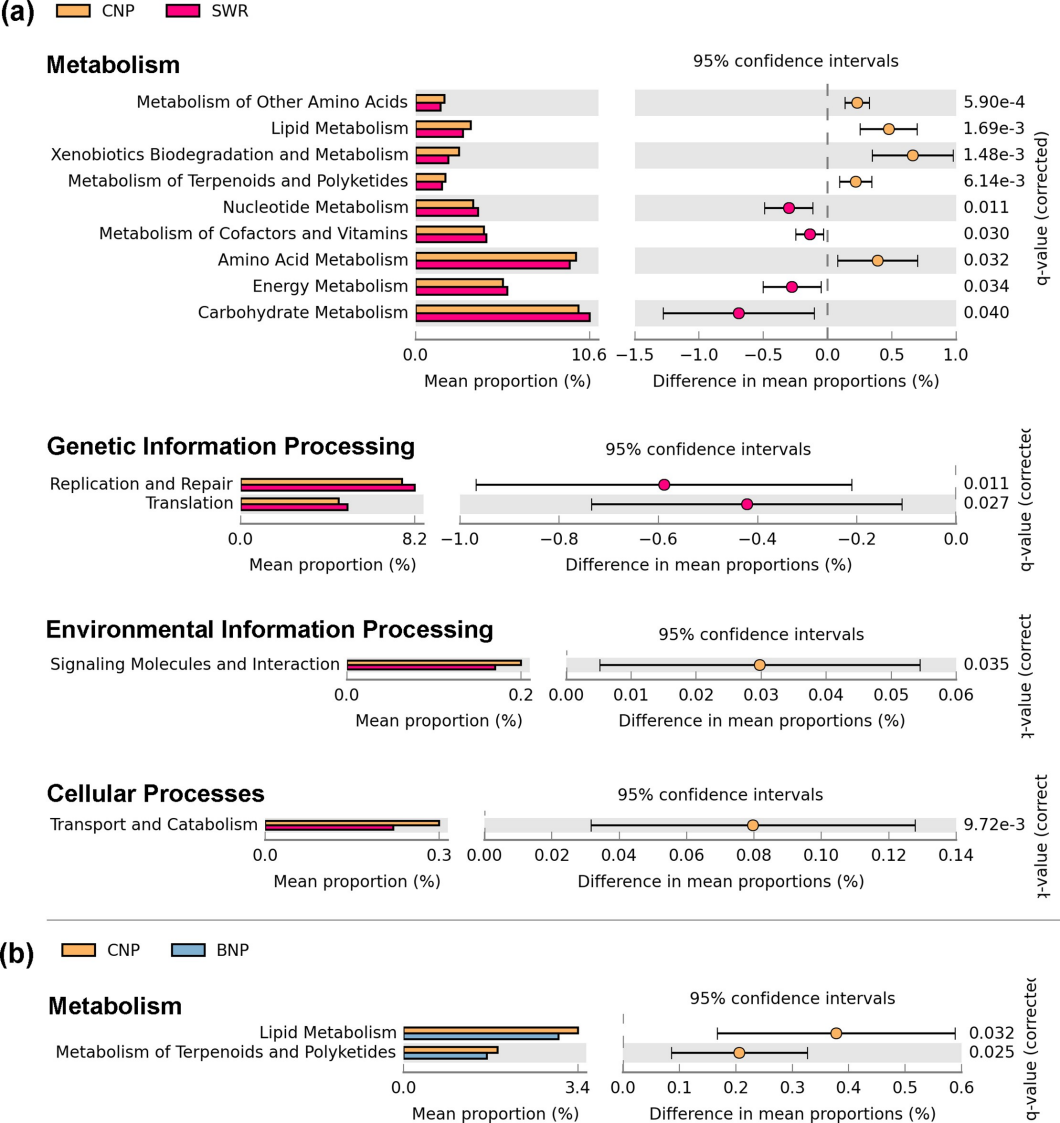
Mean proportions of predictive metabolic functional categories between tiger populations based on pair-wise comparison- functional categories are more diverse between CNP and SWR than CNP and BNP. (a) Significant functional categories identified between CNP and SWR. (b) Significant functional categories identified between CNP and BNP.

The PICRUSt analysis of all obtained OTUs using multiple group statistical ANOVA test suggested that there are notable divergences in predicted functional categories among the gut microbiota across tiger populations of CNP, BNP and SWR (*P* value < 0.05) based on eight functional categories as listed in the Table 4. Likewise, pairwise Welch’s t-test performed on the mean proportion of all the functional categories showed 13 functional pathway differences between CNP vs. SWR (*P* value < 0.05) (Fig 7A), and two in CNP vs. BNP (*P* value < 0.05) (Fig 7B). There was no significant functional pathways difference between BNP and SWR.

**Table 4:** Predictive functional profiling of microbial communities using multiple-group comparative PICRUSt analysis (*P*-value corrected < 0.05).

Functional categories	*P*-values	*P*-values (corrected)	CNP		BNP		SWR	
			mean rel. freq. (%)	std. dev. (%)	mean rel. freq. (%)	std. dev. (%)	mean rel. freq. (%)	std. dev. (%)
Metabolism of Other Amino Acids	2.46E-05	1.01E-03	1.7586	0.1840	1.6343	0.1300	1.5277	0.1463
Lipid Metabolism	5.11E-05	1.05E-03	3.3852	0.4784	3.0074	0.2283	2.9102	0.2805
Xenobiotics Biodegradation and Metabolism	2.37E-04	3.23E-03	2.6593	0.7133	2.1849	0.4225	1.9963	0.3467
Metabolism of Terpenoids and Polyketides	4.61E-04	4.72E-03	1.8282	0.2626	1.6218	0.1454	1.6080	0.1672
Transport and Catabolism	4.38E-03	2.24E-02	0.3019	0.0999	0.2527	0.0633	0.2222	0.0656
Amino Acid Metabolism	6.04E-03	2.75E-02	9.8148	0.6008	9.2886	0.6104	9.4261	0.4714
Replication and Repair	1.13E-02	4.23E-02	7.5835	0.7039	7.6596	0.7293	8.1723	0.5950
Nucleotide Metabolism	1.19E-02	4.05E-02	3.5190	0.3695	3.6291	0.3409	3.8212	0.2764

Overall significant differences were observed in predictive metabolic functions for the tiger populations studied across three protected sites within TAL. The pair-wise relationship between samples based on functional analysis corresponded to the pair-wise relation between samples based on fecal microbiota structure (PROCRUSTES, m2 = 0.72, *P* = 0.0009). This analysis further underscores the geo-location specificity with BNP samples overlapping with well separated CNP and SWR samples.

The Welch’s t-test between CNP and SWR identified higher proportions of predicted functional categories related to “amino acid metabolism”, “lipid metabolism”, “xenobiotics biodegradation and metabolism”, and “metabolism of terpenoides and polyketides” in CNP samples. While SWR samples had higher proportions of “energy metabolism”, “carbohydrate metabolism”, “metabolism of cofactors and vitamins” and “nucleotide metabolism” categories in comparison with CNP (Fig 7A). Similarly, the Welch’s t-test between CNP and BNP identified lower proportions of “lipid metabolism” and “metabolism of terpenoids and polyketides” in BNP samples than the CNP samples.

## Discussion

We found that whilst microbial alpha diversity did not differ significantly between tiger populations, phylosymbiosis among other factors most likely played a role in shaping tiger microbiota as their composition and beta diversity mirrored the host genetic patterns as observed in our previous study [32]. This supports the theory that host evolutionary background plays an important role in shaping the bacterial gut communities [17]. However, the unique compositional and functional signature of gut microbiota detected in tigers from CNP, which represents the protected site most heavily affected by human development and disturbance, although speculative, possibly shows that anthropogenic impact may also contribute to shaping microbial gut profiles in tigers by influencing biological and environmental pathways which enable bacteria within microbiota and the genes they carry to spread between different biomes [43]. Our findings have critical implications for overall tiger's health, highlighting the importance of microbiome studies in comprehensive species conservation and management efforts.

### Tiger genetics, diet or human exposure in explaining gut microbial composition

Our study shows notable differences in microbiota diversity observed between CNP and the two other protected areas, as opposed to the differences observed between BNP and SWR, giving an indication that the microbiota diversity in CNP is unique from that of BNP and SWR. This mirrors the results from our previous study where we have observed limited gene flow between tiger population from CNP with the other two protected areas (BNP and SWR) [32]. This could be due to several different factors, including higher levels of human microbiota influence on wild tigers at the CNP site. Also, habitat fragmentation, differences in diet and limited gene flow in tigers between CNP and other sites may be contributing to CNP’s unique microbiota profiles. In contrast, genetic connectivity for tigers between BNP and SWR habitats, which had more similar microbiota profiles, is supported by the known presence of wildlife corridors. Although this study is the first of its kind in wild tigers, a study in an endangered primate, red colobus monkey, showed a direct correlation between higher habitat fragmentation and reduced gut microbiota diversity, which had some profound implications on health and long-term viability of the species [12]. Low population numbers in some tiger populations and increased levels of habitat loss and fragmentation may contribute towards lowering of genetic diversity in host species, which in turn can also adversely impact gut microbiota. In the TAL region of Nepal, there has been a 97% increase in agriculture and settlement areas in the past 200 years and forested areas decreased by 47% between the 19^th^ and 20^th^ centuries [32]. In our study, we found some significant differences in gut microbial composition in three geo-spatially separated tiger population. These differences were highest between CNP and SWR tiger populations which are geographically most separated (Fig 1). Differences in habitat including differences in prey composition and tiger densities, as well as interactions across the fragmented population of these protected areas, might have an additional role in shaping such microbiota composition and diversity.

Although numerous studies have shown the effect of dietary habits on the composition of the gut microbiota [44, 45], most of them focus on structural dietary differences such as a protein-rich versus a polysaccharide-rich diet, which seems to be not relevant for a strict carnivore such as tiger. The overall gut microbiota profile in tigers that we observed in our study were similar in composition with findings reported in various other microbiome research done on mammals [19, 46–48], including carnivore species for which we have prepared a graph (S8 Fig) demonstrating their microbiota composition profiles derived from the respective research [47, 49–56] (S8 Fig). Relatively few studies on the link between gut microbiota and diet have been conducted in wild animals [17]. However, one study showed that in black howler monkeys habitat fragmentation was correlated with a less diverse diet and correspondingly less diverse gut microbiota [13].

Tiger diet composition in Nepal has only been sparsely studied, but the main prey species are chital (*Axis axis*), sambar deer (*Cervus unicolor*), hog deer (*A*. *porcinus*), barking deer (*Muntiacus muntjak*) and wild boar (*Sus scrofa*) [57]. Other species, such as swamp deer (*C*. *duvauceli*), gaur (*Bos gaurus*) and langur (*Semnopithecus entellus*), may represent a smaller part of tiger diet and also livestock may play a role in tiger diet, notably on the edges of protected areas [58, 59]. Given the habitat characteristics of the three protected areas included in this study, available diet seems to be similar across the sites. This is corroborated by a study estimating prey density as presented by Dhakal *et al*. [60]. BNP has an overall higher prey density, but in all cases chital makes up the vast majority of available tiger prey. In SWR sambar or barking deer were not detected, although they are relatively common at both the CNP and BNP sites. However, nilgai (*Boselaphus tragocamelus*) was detected relatively often at SWR, whereas this species was only seen twice during the study at Bardia and it does not occur at CNP. Although we cannot rule out that there are slight differences in dietary composition between the studied areas, we hypothesize that there are no major differences between the diets of the individual tiger populations, which would explain the observed differences in microbiota content. However, tiger diet and variation between the different tiger populations in Nepal should be subject to further investigation.

### Gut microbiota and functional metabolic implications

PICRUSt based predictive metabolic functionalities in tiger population revealed higher functional enrichment in the CNP tiger population for most categories, whereas the SWR tiger population had the lowest levels of enrichment in comparison with gut microbiota from other sites. We found significant differences in two functional categories among CNP vs BNP (Fig 7B), and 13 categories among CNP vs SWR (Fig 7A), while functional categories did not differ significantly between BNP and SWR sites (Fig 7). Predictive metabolic profiles are just rough indicators of possible functional implication of microbiota present. In conclusion, we observed that the tiger population structure, gut microbiota profile and associated functional metabolic categories are correlated, with geographically most separated CNP and SWR population having the most distinct and different host genotype and microbiota profiles.

This study further highlights the necessity of a more comprehensive systems biology based approach to assess the conservation status of the species by monitoring and maintaining genetic diversity of the host and its associated microbiota. We also encourage further investigation of various extrinsic and intrinsic factors that might influence gut microbiota and its influence on tiger health.

### Application of gut microbiota in conservation

Microbial analyses hold a great potential in uncovering information on host population dynamics, however studies in wild carnivores are scant. Such information can be used to preserve host biodiversity and develop effective conservation and management strategies. Microbiota is closely linked to health and hence, microbial phylogenies can be used as signatures of disease transmission and has potential for monitoring population health, density, movement, and dispersal [26].

## Methods

### Methods for host genetic analysis

#### Genetic database of wild tiger in Nepal and fecal DNA sampling

NTGP created Nepal’s first comprehensive tiger genetic reference database by collecting and analyzing fecal samples (*n* = 770) from the TAL (December, 2011- March, 2012) (Fig 1), which included all the known habitat of tigers in Nepal. The TAL has a sub-tropical monsoonal climate and mixed deciduous vegetation ranging from alluvial floodplain grasslands communities to Climax Sal (*Shorea robusta*) forests and includes five protected areas, among which SWR (28°50′25″N 80°13′44″E), BNP(28°23′N 81°30′E), and CNP (27°30'0.00" N 84°40'0.12" E) are the major tiger habitats (Fig 1).

Putative tiger fecal (scat) samples were collected from protected areas and connecting wildlife corridors across the TAL-Nepal [32]. Ninety-eight grid cells each measuring 15 X 15 km (225 km^2^, sampling unit) were sampled using opportunistic field surveys. A few grams from the upper surfaces of the scat were removed and stored at room temperature in sterile 2-ml vials filled with DETs buffer (dimethyl sulphoxide saline solution) [61] at 1:4 volume scat-to-solution ratio following field sampling protocols by Wultsch, Waits [62].

#### DNA extraction, species identification, and individual identification

We extracted DNA from scat samples using a commercially available QIAmp DNA Stool Kit (QIAGEN Inc., Germany) following the manufacturer’s instructions. Each batch of DNA extraction included a negative extraction control. Extracted DNA was stored at -20°C. Tigers were identified using PCR assay that used tiger specific mtDNA Cytochrome-b (*CYT-B*) primers [63]. Individual tigers were identified by microsatellite analysis using a panel of eight microsatellite markers developed from the domestic cat (*Felis catus*) and tiger genomes [64–66] as described in Thapa et al. [32].

### Gut microbiota analysis

We randomly selected a total of 70 scat samples from 32 unique individual tigers (*n* = 12, CNP; male = 9, female = 2, undetermined = 1); (*n* = 13, BNP; male = 6, female = 6, undetermined = 1); (*n* = 7, SWR; male = 4, female = 3) for gut microbiota analysis. We also selected multiple samples from 8 individual tigers (n = 2, CNP; n = 3, BNP; n = 3, SWR) (S1 Table). Soil samples were also collected from two of the study sites (*n* = 3, CNP; *n* = 5, BNP) with the goal to profile soil microbiota to assess cross-contamination between soil and fecal samples occurred.

Microbial DNA was isolated from tiger fecal and soil samples using PowerSoil DNA Isolation Kit (MoBio, Qiagen, Carlsbad, CA). DNA quality was checked by gel electrophoresis (mostly >10 kbps fragments), and DNA concentration was measured using Qubit (Invitrogen, Carlsbad, CA). We completed microbial community profiling (identification and composition) by amplifying and sequencing the hyper-variable region (V4) of the 16S rRNA from both tiger scat and soil control samples using a modified version of the protocol presented in Caporaso et. al 2012 [67], adapted for the Illumina MiSeq platform. Using a two-step polymerase chain reaction (PCR), we amplified the V4 region of the 16S rRNA using the ‘universal’ bacterial primer pairs (515F and 806R) linked to the forward and reverse Illumina flow cell adapter sequences. PCR was carried out in two steps, both using the 2X KAPA HiFi HotStart ReadyMix (KAPA Biosystems/Roche, USA) and cycling at initial denaturation at 95° C for 30sec followed by 95° C for 30sec, 55° C for30sec, 72° C for 30sec. Post cycling, samples were incubated at 72° C for 5 min, followed by a hold at 4°C. The first PCR was conducted in 25 cycles, adding a 6 bp barcode sequence to enable multiplexing. The second PCR was conducted in 8 cycles to amplify the PCR products and add the remaining full-length Illumina adapters. We purified the resulting PCR products using Agencourt AMPure XP beads (Beckman Coulter, Brea, CA), quantified with Qubit (Invitrogen, USA), normalized, and pooled all sample libraries prior to sequencing. Paired-end sequencing (2 x 300bp) was completed on Illumina MiSeq (Illumina, Inc., San Diego, CA), using a v3 600-cycle kit according to the manufacturer’s instructions.

After sequencing and de-multiplexing, we filtered all reads by quality (q>29 across 50% of the read length with no ambiguous N base calls) and length (>75 bp). A custom Perl script was written to execute several analysis modules of QIIME, version 1.9.1 [68]. First, we joined raw paired-end Illumina fastq files by fastq-join. We discarded all OTU containing less than 10 sequences. We chose the cluster centroid for each OTU as the OTU representative sequence and taxonomically assigned each sequence using homologous searches to 16S reference sequences found in the Greengenes database [69] at greater than or equal to 96% sequence identity [12, 17, 70]. To construct a phylogenetic tree of the OTU representative sequences, we aligned sequences using PyNAST, version 1.2.2 [71] against an existing alignment of the Greengenes database. Post alignment and construction of phylogenetic trees was completed using FastTree, version 2.1 [72].

Alpha-diversity, beta-diversity estimates, and relative abundance analysis of each taxonomic group were performed after rarefaction was applied with even sub-sampling of 10,000 sequences per sample. The abundances of OTUs were normalized based on proportion and OTUs with very low variability (1e^-05^) were filtered out. Microbial diversity within (alpha diversity) and between tiger subpopulations (beta diversity) were obtained and visualized with QIIME and the *phyloseq* package in R [73, 74]. We assessed alpha diversity using several metrics (Chao1, ACE, Shannon, Simpson, InvSimpson, Fisher) [39, 40, 75–77]. Statistical evaluation of differential abundance was done with F test supplemented in the mt function in *phyloseq* [74]. The resulting *P* values were adjusted for multiple comparisons using Benjamin and Hoechberg’s false discovery method (Fig 6).

To test if gut microbiota diversity were significantly differentiated across different study sites (beta diversity), we employed Permutational Multivariate Analysis of Variance (PERMANOVA) [78], canonical analysis of principal coordinates (CAP) [79], and permutational tests of homogeneity of dispersions (PERMDISP) [80]. To test for differences in both UniFrac and Bray-Curtis similarity distances, we performed a one-way PERMANOVA and used pair-wise contrasts to examine differences between sites. We analyzed compositional differences using CAP and DPCoA (detrended principal coordinate analysis) and also used the CAP discriminant analysis to validate the PERMANOVA results (i.e., how distinct was each site in multivariate space) by assessing allocation success using the ‘leave-one-out’ procedure [79]. PERMDISP was used to compare beta diversity between sites for both metrics [80] and to test if differences detected by PERMANOVA were likely due to differences in-group dispersion. All of the above analyses were conducted in PRIMER- E PERMANOVA+[81].

### Predictive metabolic functions associated with tiger gut microbiota

We used PICRUSt (Phylogenetic Investigation of Communities by Reconstruction of Unobserved States) [82] to predict functional roles played by the tiger gut microbiota communities. PICRUSt predicts metabolic and functional profiles of a microorganism based on known functional roles of its closely related microorganism [82]. It utilizes existing information from Integrated Microbial Genomes (IMG) database [83], which contains annotation of gene content and 16S copy number data of reference bacterial and archaeal genomes. Then by implementing extended ancestral-state reconstruction algorithm, the taxonomic composition and phylogenetic information of the observed OTUs are used in estimating the comprehensive metagenome of the microbiota community classifying their metabolic and functional categories in the KEGG Orthology (KO) classification scheme [84]. The PICRUSt predictions were subjected to statistical analyses with Statistical Analysis of Metagenomic Profiles (STAMP) [85] for identifying and characterizing significant functional categories across three subpopulations CNP, BNP, and SWR. We conducted multiple group statistical tests with ANOVA and pair-wise statistical tests using Welch’s t-test to test for statistical differences in mean proportion of functional categories among subpopulations. The *P*-value was adjusted by applying the Benjamini-Hochberg false discovery rate (FDR) method to correct for multiple hypotheses testing. We conducted Procrustes [86] analysis using QIIME to test correlations on beta-diversity obtained for gut microbiota and predictive microbiota functionality contents using Bray-Curtis distance metrics.

## Supporting information

S1 Fig Rarefaction curves for Observed OTUs richness indices of microbiota. (DOCX)

S2 Fig Gut microbiota profile of tiger A from multiple scat samples collected from the CNP. (DOCX)

S3 Fig Gut microbiota profile of tiger B from multiple scat samples collected from the CNP. (DOCX)

S4 Fig Gut microbiota profile of tiger C from multiple scat samples collected from the BNP. (DOCX)

S5 Fig Gut microbiota profile of tiger D from multiple scat samples collected from the BNP. (DOCX)

S6 Fig Gut microbiota profile of tiger E from multiple scat samples collected from the SWR. (DOCX)

S7 Fig Gut microbiota profile of tiger F from multiple scat samples collected from the SWR. (DOCX)

S8 Fig Comparative microbiota profiles in carnivore gut and soil samples compiled from various microbiome research. Representation of microbial biodiversity found in various carnivore species, including environmental samples (soil). For the soil and Bengal tiger, we used data from our current study. The data for Dhole1 [54], Dhole2 [49], Wolf [56], Giant panda [55], Snow leopard [47], Antarctic seals [52], Domestic cat1 [53], Domestic cat2 [50] and Cheetah [51] were compiled from other published studies.
(DOCX)

S1 Table DNA Sequence read counts of samples based on 16S microbial marker, along with corresponding sample details on sex, location, individual ID and genotype data. (DOCX)
