# Supplementary figures and images for: Gut microbiota and their putative metabolic functions in fragmented Bengal tiger population of Nepal
Source: PLoS One. 2019 Aug 29;14(8):e0221868. doi: 10.1371/journal.pone.0221868 (PMC6715213; doi:10.1371/journal.pone.0221868)

**S1 Fig. Rarefaction curves for Observed OTUs richness indices of microbiota**


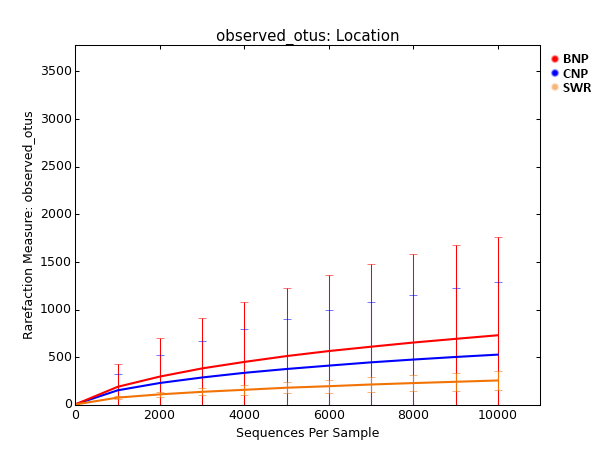

Supplement: S1 Fig — (DOCX) [file pone.0221868.s001.docx]
