# Supplementary material for: Gut microbiota and their putative metabolic functions in fragmented Bengal tiger population of Nepal
Source: PLoS One. 2019 Aug 29;14(8):e0221868. doi: 10.1371/journal.pone.0221868 (PMC6715213; doi:10.1371/journal.pone.0221868)

**S4 Fig. Gut microbiota profile of tiger C from multiple scat samples collected from the Bardia National Park**


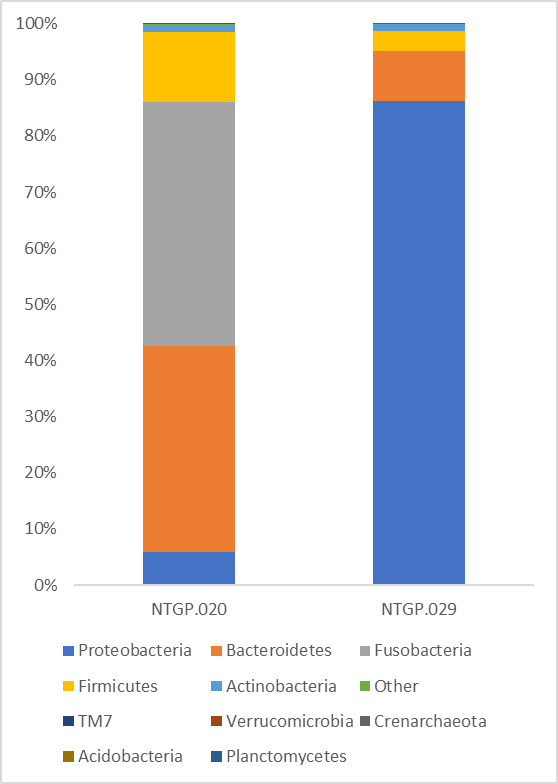

Supplement: S4 Fig — (DOCX) [file pone.0221868.s004.docx]
