# Supplementary material for: Gut microbiota and their putative metabolic functions in fragmented Bengal tiger population of Nepal
Source: PLoS One. 2019 Aug 29;14(8):e0221868. doi: 10.1371/journal.pone.0221868 (PMC6715213; doi:10.1371/journal.pone.0221868)

**S8 Fig. Comparative microbiota profiles in carnivore gut and soil samples**. Representation of microbial biodiversity found in various carnivore species, including environmental samples (soil). For the soil and Bengal tiger, we used data from our current study. The data for Dhole1 [[53](#_ENREF_53)], Dhole2 [[48](#_ENREF_48)], Wolf [[55](#_ENREF_55)], Giant panda [[54](#_ENREF_54)], Snow leopard [[46](#_ENREF_46)], Antarctic seals [[51](#_ENREF_51)], Domestic cat1 [[52](#_ENREF_52)], Domestic cat2 [[49](#_ENREF_49)] and Cheetah [[50](#_ENREF_50)] were compiled from other published studies.


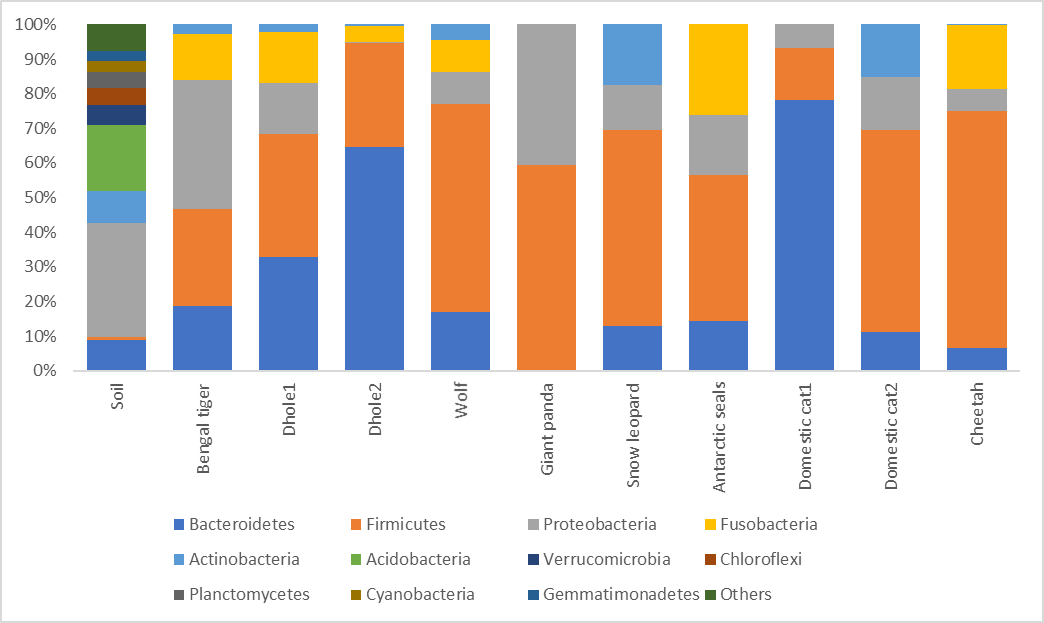

Supplement: S8 Fig — Representation of microbial biodiversity found in various carnivore species, including environmental samples (soil). For the soil and Bengal tiger, we used data from our current study. The data for Dhole1 [54], Dhole2 [49], Wolf [56], Giant panda [55], Snow leopard [47], Antarctic seals [52], Domestic cat1 [53], Domestic cat2 [50] and Cheetah [51] were compiled from other published studies. (DOCX) [file pone.0221868.s008.docx]
