# Supplementary material for: Gut microbiota and their putative metabolic functions in fragmented Bengal tiger population of Nepal
Source: PLoS One. 2019 Aug 29;14(8):e0221868. doi: 10.1371/journal.pone.0221868 (PMC6715213; doi:10.1371/journal.pone.0221868)
Supplement: S1 Table — (DOCX) [file pone.0221868.s009.docx]

S1 Table. DNA sequence read counts of samples based on 16S microbial marker, along with corresponding sample details on sex, location, individual ID and genotype data. (NA=Not Available)

| **SampleID** | **NGS Read counts** | **Sex** | **Sample type** | **Location** | **Individual No.** | **Microsatellite markers** | | | | | | | | | | | | | | | | | | | | |
| --- | --- | --- | --- | --- | --- | --- | --- | --- | --- | --- | --- | --- | --- | --- | --- | --- | --- | --- | --- | --- | --- | --- | --- | --- | --- | --- |
|  |  |  |  |  |  | **FCA205** | | **FCA391** | | **PttD5** | | **FCA232** | | **FCA304** | | **FCA043** | | **F53** | | **F85** | | **FCA441** | | **PttA2** | |  |
| NTGP.0328 | 33124 | Male | Scat | CNP | 1 | NA | NA | 144 | 152 | 207 | 207 | 99 | 101 | 125 | 125 | 115 | 115 | 164 | 172 | 165 | 165 | 111 | 119 | NA | NA |  |
| NTGP.0328 | 39441 |  |  |  |  | NA | NA | 144 | 152 | 207 | 207 | 99 | 101 | 125 | 125 | 115 | 115 | 164 | 172 | 165 | 165 | 111 | 119 | NA | NA |  |
| NTGP.0329 | 58070 |  |  |  |  | NA | NA | 144 | 152 | 199 | 207 | 99 | 101 | 125 | 125 | 115 | 115 | 164 | 172 | 157 | 165 | 111 | 119 | NA | NA |  |
| NTGP.0334 | 58651 |  |  |  |  | 108 | 110 | 144 | 152 | 207 | 207 | 99 | 101 | 125 | 125 | 113 | 115 | 164 | 172 | 165 | 165 | 111 | 119 | NA | NA |  |
| NTGP.0398 | 46531 |  |  |  |  | NA | NA | 144 | 152 | 207 | 207 | 99 | 101 | 125 | 125 | 115 | 115 | 164 | 172 | 165 | 165 | 111 | 119 | NA | NA |  |
| NTGP.0402 | 2588 |  |  |  |  | 106 | 108 | 144 | 152 | 207 | 207 | 99 | 101 | 125 | 125 | 115 | 115 | 164 | 172 | 165 | 165 | 111 | 119 | NA | NA |  |
| NTGP.0403 | 48848 |  |  |  |  | 108 | 110 | 144 | 152 | 207 | 207 | 99 | 101 | 125 | 125 | 115 | 115 | 164 | 172 | 165 | 165 | 111 | 119 | NA | NA |  |
| NTGP.0475 | 54578 |  |  |  |  | NA | NA | 144 | 152 | 207 | 207 | 99 | 101 | 125 | 125 | 115 | 115 | NA | NA | NA | NA | 111 | 119 | NA | NA |  |
| NTGP.0496 | 43532 |  |  |  |  | NA | NA | 144 | 152 | 207 | 207 | 99 | 101 | 125 | 125 | 115 | 115 | 164 | 172 | 165 | 165 | 111 | 119 | NA | NA |  |
| NTGP.0496 | 45426 |  |  |  |  | NA | NA | 144 | 152 | 207 | 207 | 99 | 101 | 125 | 125 | 115 | 115 | 164 | 172 | 165 | 165 | 111 | 119 | NA | NA |  |
| NTGP.0498 | 9164 |  |  |  |  | NA | NA | 144 | 152 | 207 | 207 | 99 | 101 | 125 | 125 | 115 | 115 | 164 | 172 | 165 | 165 | 111 | 119 | NA | NA |  |
| NTGP.0300 | 48488 | Male | Scat | CNP | 2 | 108 | 110 | 144 | 156 | 199 | 207 | 99 | 101 | 125 | 139 | 115 | 115 | 164 | 176 | 165 | 165 | 111 | 111 | NA | NA |  |
| NTGP.0302 | 51724 |  |  |  |  | NA | NA | 144 | 156 | 199 | 207 | 99 | 101 | 125 | 139 | 115 | 115 | 164 | 176 | 165 | 165 | 111 | 111 | NA | NA |  |
| NTGP.0303 | 53156 |  |  |  |  | NA | NA | 144 | 156 | 199 | 207 | 99 | 101 | 125 | 139 | 115 | 115 | 164 | 176 | 165 | 165 | 111 | 111 | NA | NA |  |
| NTGP.0304 | 46115 |  |  |  |  | NA | NA | 144 | 156 | 199 | 207 | 99 | 101 | 125 | 139 | 115 | 115 | 164 | 176 | 165 | 165 | 111 | 111 | NA | NA |  |
| NTGP.0382 | 51456 |  |  |  |  | NA | NA | 144 | 156 | 199 | 207 | 99 | 101 | 125 | 139 | 115 | 115 | 164 | 176 | 165 | 165 | 111 | 111 | NA | NA |  |
| NTGP.0383 | 48419 |  |  |  |  | 108 | 108 | 144 | 156 | 199 | 207 | 99 | 101 | 125 | 139 | 115 | 115 | 164 | 176 | 165 | 165 | 111 | 111 | NA | NA |  |
| NTGP.0386 | 39377 |  |  |  |  | 108 | 110 | 144 | 156 | 199 | 207 | 99 | 101 | 125 | 139 | 115 | 115 | 164 | 176 | 165 | 165 | 111 | 111 | NA | NA |  |
| NTGP.0447 | 45134 |  |  |  |  | 108 | 110 | 144 | 156 | 199 | 207 | 99 | 101 | 125 | 139 | 115 | 115 | 164 | 176 | 165 | 165 | 111 | 111 | NA | NA |  |
| NTGP.0002 | 45836 | Female | Scat | BNP | 3 | NA | NA | 152 | 156 | 207 | 211 | 99 | 101 | 121 | 125 | 115 | 115 | 172 | 176 | 165 | 165 | 107 | 115 | 186 | 186 |  |
| NTGP.0020 | 56030 |  |  |  |  | 100 | 108 | 152 | 156 | 207 | 211 | 101 | 101 | 121 | 125 | 115 | 115 | 172 | 176 | 165 | 165 | 107 | 115 | 186 | 186 |  |
| NTGP.0021 | 50774 |  |  |  |  | NA | NA | 152 | 156 | 207 | 211 | 101 | 101 | 121 | 125 | 115 | 115 | 176 | 176 | 165 | 165 | 107 | 115 | 186 | 186 |  |
| NTGP.0029 | 50551 |  |  |  |  | NA | NA | 152 | 156 | 207 | 211 | 99 | 101 | 121 | 125 | 115 | 115 | 172 | NA | 165 | 165 | 107 | 115 | 186 | 186 |  |
| NTGP.0037 | 59238 | Female | Scat | BNP | 4 | NA | NA | 152 | 152 | 207 | 211 | 101 | 101 | 121 | 125 | 115 | 115 | NA | NA | 165 | 165 | 115 | 119 | 186 | 186 |  |
| NTGP.0749 | 59977 |  |  |  |  | 106 | 110 | 152 | 152 | 207 | 211 | 101 | 101 | 121 | 125 | 115 | 115 | 160 | 172 | NA | 165 | 115 | 119 | 186 | 186 |  |
| NTGP.0751 | 43645 |  |  |  |  | 106 | 110 | 152 | 152 | 207 | 211 | 101 | 101 | 121 | 125 | 115 | 115 | 160 | 172 | 165 | 165 | 115 | 119 | 186 | 186 |  |
| NTGP.0752 | 54471 |  |  |  |  | NA | NA | 152 | 152 | 207 | 211 | 101 | NA | 121 | 125 | 115 | 115 | NA | NA | 165 | 165 | 115 | 119 | 186 | 186 |  |
| NTGP.0139 | 49530 | Male | Scat | SWR | 5 | 106 | 108 | 156 | 156 | 199 | 211 | 101 | 101 | 121 | 121 | 119 | 119 | 160 | 180 | 161 | 165 | 119 | 119 | 186 | 194 |  |
| NTGP.0139 | 51795 |  |  |  |  | 106 | 108 | 156 | 156 | 199 | 211 | 101 | 101 | 121 | 121 | 119 | 119 | 160 | 180 | 161 | 165 | 119 | 119 | 186 | 194 |  |
| NTGP.0247 | 25707 |  |  |  |  | 106 | 108 | 156 | 156 | 199 | 211 | 101 | 101 | 121 | 121 | 119 | 119 | 160 | 180 | 161 | 165 | 119 | 119 | 186 | 194 |  |
| NTGP.0247 | 40798 |  |  |  |  | 106 | 108 | 156 | 156 | 199 | 211 | 101 | 101 | 121 | 121 | 119 | 119 | 160 | 180 | 161 | 165 | 119 | 119 | 186 | 194 |  |
| NTGP.0248 | 59134 |  |  |  |  | 106 | 108 | 156 | 156 | 199 | 211 | 101 | 101 | 121 | 121 | 119 | 119 | 160 | 180 | 161 | 165 | 119 | 119 | 186 | 194 |  |
| NTGP.0249 | 30855 |  |  |  |  | 106 | 108 | 156 | 156 | 199 | 211 | 101 | 101 | 121 | 121 | 119 | 119 | 160 | 180 | 161 | 165 | 119 | 119 | 186 | 194 |  |
| NTGP.0253 | 45176 |  |  |  |  | 106 | 108 | 156 | 156 | 199 | 211 | 101 | 101 | 121 | 121 | 119 | 119 | 160 | 180 | 161 | 165 | 111 | 119 | 186 | 194 |  |
| NTGP.0271 | 59943 |  |  |  |  | 106 | 108 | 156 | 156 | 199 | 211 | 101 | 101 | 121 | 121 | 119 | 119 | 160 | 180 | 161 | 165 | 119 | 119 | 186 | 194 |  |
| NTGP.0206 | 53711 | Female | Scat | SWR | 6 | 106 | 108 | 152 | 152 | 211 | 211 | 101 | 101 | 125 | 125 | 115 | 123 | 164 | 164 | 165 | 165 | 115 | 123 | NA | NA |  |
| NTGP.0234 | 48803 |  |  |  |  | 108 | 108 | 152 | 152 | 211 | 211 | 101 | 101 | 125 | 125 | 115 | 123 | 164 | 164 | 165 | 165 | 115 | 123 | 188 | 188 |  |
| NTGP.0234 | 47121 |  |  |  |  | 108 | 108 | 152 | 152 | 211 | 211 | 101 | 101 | 125 | 125 | 115 | 123 | 164 | 164 | 165 | 165 | 115 | 123 | 188 | 188 |  |
| NTGP.0238 | 37907 |  |  |  |  | 108 | 108 | 152 | 152 | 211 | 211 | 101 | 101 | 125 | 125 | 115 | 123 | 164 | 164 | 165 | 165 | 115 | 123 | 186 | 186 |  |
| NTGP.0250 | 55695 |  |  |  |  | 108 | 108 | 152 | 152 | 211 | 211 | 101 | 101 | 125 | 125 | 115 | 123 | 164 | 164 | 165 | 165 | 115 | 123 | NA | NA |  |
| NTGP.0264 | 46512 |  |  |  |  | 108 | 108 | 152 | 152 | 211 | 211 | 101 | 101 | 125 | 125 | 115 | 123 | 164 | 164 | 165 | 165 | 115 | 123 | 186 | 186 |  |
| NTGP.0027 | 24589 | Male | Scat | BNP | 7 | 98 | 98 | NA | NA | 233 | 244 | NA | NA | 115 | 115 | 115 | 115 | 152 | 172 | 157 | 165 | 99 | 103 | 180 | 186 |  |
| NTGP.0050 | 29051 | Female | Scat | BNP | 8 | 110 | 110 | 152 | 152 | 207 | 211 | 101 | 107 | 121 | 125 | 115 | 123 | NA | NA | 165 | 165 | 107 | 119 | 186 | 186 |  |
| NTGP.0060 | 44208 | NA | Scat | BNP | 9 | 110 | 110 | 152 | 152 | 199 | 207 | 101 | 101 | 125 | 125 | 115 | 115 | NA | 172 | 157 | 165 | 107 | 119 | 186 | 186 |  |
| NTGP.0087 | 37166 | Male | Scat | BNP | 10 | 100 | 106 | 156 | 156 | 199 | 211 | NA | 101 | 121 | 125 | 115 | 129 | 164 | 176 | 165 | 165 | 115 | 119 | 186 | 186 |  |
| NTGP.0094 | 25343 | Male | Scat | BNP | 11 | 108 | 110 | 152 | 152 | 207 | 207 | 101 | 101 | 125 | 125 | 115 | 117 | 160 | 164 | 157 | 165 | 119 | 119 | 186 | 186 |  |
| NTGP.0116 | 8336 | Female | Scat | BNP | 12 | 108 | 110 | 152 | 152 | 207 | 207 | 101 | 101 | 125 | 125 | 115 | 117 | 160 | 164 | 157 | 161 | 119 | 119 | 186 | 186 |  |
| NTGP.0118 | 38005 |  |  |  |  | 108 | 110 | 152 | 152 | 207 | 207 | 101 | 101 | 125 | 125 | 115 | 117 | 160 | 164 | 157 | 165 | 119 | 119 | 186 | 186 |  |
| NTGP.0105 | 45610 | Female | Scat | BNP | 13 | NA | NA | 152 | 152 | 207 | 207 | 101 | 101 | 125 | 125 | 117 | 123 | 160 | 172 | 157 | 165 | 119 | 119 | 186 | 186 |  |
| NTGP.0114 | 34836 | Male | Scat | BNP | 14 | 108 | 108 | 152 | 152 | 207 | 207 | 101 | 101 | 125 | 125 | 117 | 123 | 160 | 160 | 157 | 165 | 111 | 119 | 186 | 186 |  |
| NTGP.0313 | 45263 | Male | Scat | CNP | 15 | 108 | 108 | NA | NA | 203 | 207 | 101 | 101 | 125 | 139 | 115 | 119 | 164 | 172 | 161 | 161 | 111 | 115 | 186 | 186 |  |
| NTGP.0316 | 95553 | Male | Scat | CNP | 16 | 108 | 108 | 152 | 152 | 207 | 207 | 99 | 101 | 125 | 125 | 115 | 117 | 160 | 172 | 157 | 165 | 115 | 119 | 186 | 186 |  |
| NTGP.0353 | 44704 | Male | Scat | CNP | 17 | 108 | 108 | 152 | 152 | 203 | 207 | 101 | 103 | 125 | 125 | 115 | 115 | 160 | 172 | 165 | 169 | 107 | 115 | 188 | 188 |  |
| NTGP.0372 | 31501 | Female | Scat | CNP | 18 | NA | NA | 144 | 152 | 207 | 207 | 101 | 101 | 125 | 125 | 115 | 115 | 164 | 180 | 165 | 165 | 111 | 111 | NA | NA |  |
| NTGP.0379 | 47917 | Male | Scat | CNP | 19 | NA | NA | 144 | NA | 207 | 207 | 99 | 101 | 125 | 125 | 115 | 115 | 176 | 176 | 165 | 169 | 111 | 115 | NA | NA |  |
| NTGP.0453 | 36891 | Male | Scat | CNP | 20 | NA | NA | 148 | 156 | 199 | 207 | NA | NA | 125 | 139 | 115 | 115 | 164 | 168 | 165 | 169 | 111 | 111 | NA | NA |  |
| NTGP.0480 | 12077 | NA | Scat | CNP | 21 | NA | NA | 144 | 152 | 207 | 207 | 99 | 101 | 125 | 125 | 115 | 115 | 176 | 180 | 165 | 165 | 115 | 119 | NA | NA |  |
| NTGP.0574 | 37964 | Female | Scat | CNP | 22 | NA | NA | 152 | 152 | 207 | 207 | NA | NA | 125 | 125 | 115 | 115 | 176 | 176 | NA | NA | 115 | 119 | 186 | 186 |  |
| NTGP.0609 | 42744 | Male | Scat | CNP | 23 | NA | NA | 148 | 152 | 207 | 207 | 101 | 107 | 121 | 125 | 123 | 123 | 176 | 176 | 153 | 165 | 111 | 115 | NA | NA |  |
| NTGP.0642 | 37768 | Male | Scat | CNP | 24 | NA | NA | 144 | 152 | 199 | 207 | NA | 101 | 125 | 125 | 115 | 115 | 176 | 176 | 165 | 165 | 115 | 119 | NA | NA |  |
| NTGP.0670 | 25200 | Male | Scat | BNP | 25 | NA | NA | 152 | 156 | 199 | 207 | 99 | 101 | 125 | 139 | 115 | 129 | NA | NA | 165 | 169 | 111 | 115 | NA | NA |  |
| NTGP.0693 | 39719 | Female | Scat | BNP | 26 | NA | NA | 152 | 152 | 207 | 207 | 101 | 101 | 121 | 123 | 115 | 115 | 176 | 180 | 153 | 153 | 115 | 115 | NA | NA |  |
| NTGP.0722 | 39707 | Male | Scat | BNP | 27 | NA | NA | 148 | 148 | 207 | 207 | 99 | 107 | 121 | 125 | 123 | 129 | 172 | 172 | 165 | 169 | 111 | 115 | NA | NA |  |
| NTGP.0148 | 40195 | Female | Scat | SWR | 28 | 106 | 108 | 156 | 156 | 199 | 211 | 101 | 101 | 121 | 121 | 115 | 115 | 160 | 176 | 157 | 161 | 115 | 119 | 186 | 196 |  |
| NTGP.0196 | 50590 |  |  |  |  | 106 | 108 | 156 | 156 | 199 | 211 | 101 | 101 | 121 | 121 | 115 | 115 | 160 | 176 | 157 | 161 | 115 | 119 | 186 | 196 |  |
| NTGP.0161 | 30575 | Male | Scat | SWR | 29 | 108 | 108 | NA | 156 | 211 | 211 | 101 | 101 | 121 | 121 | 115 | 119 | NA | NA | 157 | 165 | 115 | 123 | 186 | 186 |  |
| NTGP.0205 | 46995 | Male | Scat | SWR | 30 | 106 | 108 | 152 | 156 | 199 | 211 | 101 | 101 | 125 | 125 | 115 | 115 | 164 | 164 | 161 | 161 | 115 | 115 | 186 | 186 |  |
| NTGP.0210 | 1614 | Male | Scat | SWR | 31 | 108 | 108 | 152 | 156 | 199 | 211 | 101 | 101 | 125 | 125 | 115 | 115 | 176 | 176 | NA | 165 | 115 | 123 | 186 | 196 |  |
| NTGP.0212 | 54706 | Female | Scat | SWR | 32 | 108 | 108 | 152 | 156 | 211 | 211 | 101 | 101 | 125 | 125 | 115 | 115 | 164 | 164 | 161 | 165 | 123 | 123 | 196 | 196 |  |
| NTGP.0215 | 39956 |  |  |  |  | 108 | 108 | 152 | 156 | 211 | 211 | 101 | 101 | 125 | 125 | 115 | 115 | 164 | 164 | 161 | 165 | 123 | 123 | 196 | 196 |  |
